# Young age and adequate BCG are key factors for optimal BCG treatment efficacy in non-muscle-invasive bladder cancer

**DOI:** 10.1007/s00345-024-05218-4

**Published:** 2024-09-27

**Authors:** Kang Liu, Rossella Nicoletti, Hongda Zhao, Xuan Chen, Hongwei Wu, Chi-Ho Leung, David D’Andrea, Ekaterina Laukhtina, Francesco Soria, Andrea Gallioli, Marcelo Langer Wroclawski, Daniele Castellani, Vineet Gauhar, Juan Gomez Rivas, Dmitry Enikeev, Paolo Gontero, Shahrokh F. Shariat, Peter Ka-Fung Chiu, Chi-Fai Ng, Jeremy Yuen-Chun Teoh

**Affiliations:** 1 S. H. Ho Urology Centre, Faculty of Medicine, Department of Surgery, The Chinese University of Hong Kong, 4/F LCW Clinical Sciences Building, Prince of Wales Hospital, Shatin, Hong Kong; 2 Department of Experimental and Clinical Biomedical Science, University of Florence, Florence, Italy; 3 Department of Urology, Comprehensive Cancer Center, Medical University of Vienna, Vienna, Austria; 4 Urothelial Cancer Working Group, European Association of Urology-Young Academic Urologists (EAU-YAU), Amsterdam, Netherlands; 5 Division of Urology, Department of Surgical Sciences, San Giovanni Battista Hospital, University of Studies of Torino, 10024 Turin, Italy; 6 Department of Urology, Fundació Puigvert, Autonoma University of Barcelona, Barcelona, Spain; 7 Department of Urology, Hospital Israelita Albert Einstein, São Paulo, Brazil; 8 Department of Urology, Hospital Beneficencia Portuguesa de Sao Paulo, São Paulo, Brazil; 9 Department of Urology, Faculdade de Medicina Do ABC, Santo André, Brazil; 10 Urology Unit, Azienda Ospedaliero-Universitaria Delle Marche, Università Politecnica Delle Marche, Ancona, Italy; 11 Department of Urology, Ng Teng Fong General Hospital, National University Health System, Singapore, Singapore; 12 Department of Urology, Clinico San Carlos University Hospital, Madrid, Spain; 13 Karl Landsteiner Institute of Urology and Andrology, Vienna, Austria; 14 Division of Urology, Rabin Medical Center, Petah Tikva, Israel; 15 Department of Urology, Città Della Salute E Della Scienza, University of Torino School of Medicine, Turin, Italy; 16 2nd Faculty of Medicine, Hospital Motol, Department of Urology, Charles University, Prague, Czech Republic; 17 Department of Urology, Weill Cornell Medical College, New York, NY USA; 18 Department of Urology, University of Texas Southwestern, Dallas, TX USA; 19 Hourani Center for Applied Scientific Research, Al-Ahliyya Amman University, Amman, Jordan; 20 Cancer Prognostics and Health Outcomes Unit, University of Montreal Health Centre, Montreal, Canada; 21 Research Center for Evidence-Based Medicine, Iranian EBM Center: A Joanna Briggs Institute Center of Excellence, Tabriz University of Medical Sciences, Tabriz, Iran

**Keywords:** Non, Muscle, Invasive bladder cancer, Age, Overall survival, Cancer, Specific survival, Recurrence, Free survival, Progression, Free survival

## Abstract

**Objective:**

To investigate the impact of ageing on survival outcomes in Bacillus Calmette–Guérin (BCG) treated non-muscle invasive bladder cancer (NMIBC) patients and its synergy with adequate BCG treatment.

**Method:**

Patients with NMIBC who received BCG treatment from 2001 to 2020 were divided into group 1 (< = 70 years) and group 2 (> 70 years). Overall Survival (OS), Cancer-Specific Survival (CSS), Recurrence-Free Survival (RFS), and Progression-Free Survival (PFS) were analyzed using the Kaplan–Meier method. Multivariable Cox regression analysis was used to adjust potential confounding factors and to estimate Hazard Ratio (HR) and 95% Confidence Interval (CI). Subgroup analysis was performed according to adequate versus inadequate BCG treatment.

**Results:**

Overall, 2602 NMIBC patients were included: 1051 (40.4%) and 1551 (59.6%) in groups 1 and 2, respectively. At median follow-up of 11.0 years, group 1 (< = 70 years) was associated with better OS, CSS, and RFS, but not PFS as compared to group 2 (> 70 years). At subgroup analysis, patients in group 1 treated with adequate BCG showed better OS, CSS, RFS, and PFS as compared with inadequate BCG treatment in group 2, while patients in group 2 receiving adequate BCG treatment had 41% less progression than those treated with inadequate BCG from the same group.

**Conclusions:**

Being younger (< = 70 years) was associated with better OS, CSS, and RFS, but not PFS. Older patients (> 70 years) who received adequate BCG treatment had similar PFS as those younger with adequate BCG treatment.

**Supplementary Information:**

The online version contains supplementary material available at 10.1007/s00345-024-05218-4.

## Introduction

By 2040, approximately 991,000 new bladder cancer (BC) cases and 397,000 BC deaths will occur globally [1]. The average age of BC diagnosis is 70 years [2]. Adjuvant intravesical Bacillus Calmette–Guérin (BCG) immunotherapy is the standard first-line treatment of intermediate- and high-risk non-muscle-invasive bladder cancer (NMIBC) patients after complete transurethral resection of bladder tumour (TURBT) [3–5]. Although the mechanism of action has not yet been fully explained, it is considered based on the internalization of BCG by cancer cells and activation of the host immune system [6, 7].

Population ageing is a pervasive global demographic trend. Despite the decreasing trends of bladder cancer incidence in Hong Kong [8], the number of elderly individuals will increase from 1.45 million to 2.74 million by the middle of this century [9]. Previous studies suggested immunosenescence of innate and adaptive immunity with age [10]: while ageing attenuates the ability of immune defense, immune surveillance and immune homeostasis of the immune system, the ability to respond to intravesical BCG treatment may decrease, leading to inferior efficacy and worse survival outcomes [11]. However, poor evidences exist in this regard. On the other hand, adequate intravesical BCG immunotherapy is crucial in patients with intermediate- and high-risk NMIBC [12]. But, the impact of age on the treatment efficacy of adequate BCG remains unclear.

We aim to assess the impact of age and its synergy with adequate BCG treatment on long-term oncological outcomes within a large retrospective territory-wide database of NMIBC patients treated with adjuvant intravesical BCG [12].

## Method

### Data source and cohort building

Data from NMIBC patients treated with adjuvant BCG treatment were retrospectively collected; the complete identification and retrieval processes was reported previously [12]. In brief, according to the International Classification of Diseases, Ninth Revision, Clinical Modification (ICD-9-CM) diagnosis codes (bladder cancer: 188 and V76.3) and BCG drug codes (BCG 03, BCG 05, BCG 06, BCG 07), de-identified bladder cancer patients with intravesical BCG therapy from January 2001 to December 2020 were extracted from the Clinical Data Analysis and Reporting System (CDARS), which covered outpatient and inpatient data of approximately 80% of the 7.4 million population in Hong Kong. The exclusion criteria were a known history of or concomitant upper tract urothelial carcinoma (UTUC). The research followed ethical guidelines of the 1975 Declaration of Helsinki and was approved by the local ethics committee (approval reference number: CRE-2021.599).

Patients were divided into two groups according to their age. Group 1 included patients aged 70 years old and below, while group 2 patients aged above 70 years old. We Further stratified patients according to adequate versus inadequate BCG treatment.

### Data collection

Baseline characteristics (gender, date of birth) as well as data on comorbidities at baseline based on the Charlson Comorbidity Index (CCI) were retrieved using the ICD-9-CM diagnosis codes (Supplementary Table 1).

### Outcomes definition

Long-term survival outcomes, including Overall Survival (OS), Cancer-Specific Survival (CSS), Recurrence-Free Survival (RFS), and Progression-Free Survival (PFS) were identified. The INDEX date was the first prescription date of BCG.

OS was defined as the period from INDEX to the date of registered death or last follow-up. The CSS was determined by the registered cause of death. The recurrence date was defined as the date of the first positive TURBT after INDEX. Progression date was defined as the date of radical cystectomy or the date of first radiotherapy/ chemotherapy/ PD-L1 or PD-1 drugs administration after INDEX.

The adequate BCG therapy was defined as at least five of six induction instillations and at least one maintenance (two of three instillations) in a 6-month period [13].

### Statistical analysis

Data were analyzed using SPSS version 25.0 (SPSS, Inc., Chicago, Illinois) and R software (4.2.0; R Foundation for Statistical Computing, Vienna, Austria). Analysis was first performed comparing group 1 versus (vs.) group 2. Further subgroup analysis was performed for patients who received adequate BCG treatment. Categorical variables were presented as numbers (percentage). Qualitative and quantitative differences between groups were analyzed by the χ^2^ test for categorical parameters. Kaplan–Meier analysis was performed, and significance was determined by a log-rank test. Multivariate Cox regression analyses were performed to adjust for potential confounding factors. A *p*-value of < 0.05 was considered statistically significant.

## Result

### Baseline demographic characteristics

Overall, 2602 patients were included, 1051 (40.4%) and 1551 (59.6%) in groups 1 and 2, respectively. 55.1% of group 1 patients received adequate BCG treatment, while only 49.8% of those from group 2 (Supplementary Fig. 1).

Complete baseline characteristics are reported in supplementary Table 1. 79.8% and 77.9% of patients were males in groups 1 and 2, respectively. Regarding age-unadjusted CCI, 723/1,051 (68.8%) of group 1 only had bladder cancer (score of 2), 19.9% had a score of 3, 7.0% had a score of 4, and 4.3% had a score of 5–10. Among group 2 patients, the rates were 47.3% (score of 2), 29.8% (score of 3), 13.5% (score of 4), and 9.4% (score of 5–10). In addition, 36.4% of group 1 patients had hypertension, 21.4% had hyperlipidemia, while the rates were 63.1% and 31.8% for the group 2 group. The median follow-up of the whole cohort was 11.0 years.

Except for gender (*p* = 0.25), there were significant differences in the rates of adequate treatment (*p* = 0.01), CCI (*p* < 0.01), proportion of patients with hypertension (*p* < 0.01), and hyperlipidemia (*p* < 0.01), between two groups (Supplementary Table 2).

### Survival outcomes

Upon Kaplan–Meier analysis, while no statistically significant differences were found in PFS (*p* = 0.32), group 1 had better OS (*p* < 0.0001), CSS (*p* < 0.0001), and RFS (*p* < 0.001), as compared to group 2 (Supplementary Fig. 2). At Multivariable Cox regression analysis, younger age (group 1) was an independent protective factor for OS (HR: 0.29, 95% CI 0.25–0.35, *p* < 0.001), CSS (HR: 0.51, 95% CI 0.36–0.72, *p* < 0.001), and RFS (HR: 0.82, 95% CI 0.71–0.95, *p* < 0.01), but not for PFS (HR: 1.09, 95% CI 0.82–1.45, *p* = 0.55).

Adequate BCG treatment was an independent protective factor for OS (HR: 0.78, 95% CI 0.68–0.88, *p* < 0.001), CSS (HR: 0.63, 95% CI 0.46–0.85, *p* < 0.01), RFS (HR: 0.81, 95% CI 0.70–0.92, *p* < 0.01), and PFS (HR: 0.55, 95% CI 0.41–0.72, *p* < 0.001). Male gender was an independent risk factor for OS (HR: 1.35, 95% CI 1.14–1.58, *p* < 0.001), RFS (HR: 1.24, 95% CI 1.04–1.47, *p* = 0.02), and PFS (HR: 1.91, 95% CI 1.29–2.85, *p* < 0.01). Compared to patients who only had bladder cancer (score of 2), higher CCI score was associated with worse OS (HR: 1.46–2.72, *p* < 0.001), but not CSS, RFS, and PFS. In addition, hypertension was associated with worse RFS (HR: 1.18, 95%CI 1.01–1.38, *p* = 0.04), while hyperlipidemia was associated with better OS (HR: 0.70, 95%CI 0.60–0.83, *p* < 0.001) (Table 1).

**Table 1:** Multivariable Cox regression analysis

Variables	Overall survival		Cancer-specific survival		Recurrence-free survival		Progression-free survival	
	HR (95%CI)	*p* value	HR (95%CI)	*p* value	HR (95%CI)	*p* value	HR (95%CI)	*p* value
*Age*								
Group 2 (> 70y)	Ref		Ref		Ref		Ref	
Group 1 (< = 70y)	**0.29 (0.25–0.35)**	< 0.001	**0.51 (0.36–0.72)**	< 0.001	**0.82 (0.71–0.95)**	< 0.01	1.09 (0.82–1.45)	0.55
*Adequate BCG*								
No	Ref		Ref		Ref		Ref	
Yes	**0.78 (0.68–0.88)**	< 0.001	**0.63 (0.46–0.85)**	< 0.01	**0.81 (0.70–0.92)**	< 0.01	**0.55 (0.41–0.72)**	< 0.001
*Gender*								
Female	Ref		Ref		Ref		Ref	
Male	**1.35 (1.14–1.58)**	< 0.001	1.04 (0.73–1.49)	0.93	**1.24 (1.04–1.47)**	0.02	**1.91 (1.29–2.85)**	< 0.01
*Charlson Comorbidity Index*								
2	Ref		Ref		Ref		Ref	
3	**1.46 (1.25–1.71)**	< 0.001	0.99 (0.68–1.43)	0.94	1.03 (0.87–1.21)	0.77	0.81 (0.57–1.15)	0.24
4	**2.08 (1.69–2.57)**	< 0.001	1.17 (0.69–1.99)	0.55	0.95 (0.74–1.21)	0.67	0.91 (0.55–1.50)	0.71
5–10	**2.72 (2.17–3.40)**	< 0.001	1.22 (0.65–2.29)	0.54	1.04 (0.78–1.38)	0.82	0.83 (0.44–1.58)	0.57
*Hypertension*								
No	Ref		Ref		Ref		Ref	
Yes	1.02 (0.88–1.18)	0.81	1.16 (0.83–1.62)	0.40	**1.18 (1.01–1.38)**	0.04	0.99 (0.73–1.35)	0.96
*Hyperlipidemia*								
No	Ref		Ref		Ref		Ref	
Yes	**0.70 (0.60–0.83)**	< 0.001	0.69 (0.46–1.04)	0.08	0.88 (0.74–1.05)	0.16	0.79 (0.54–1.16)	0.23

### Subgroup analysis for adequate BCG treatment

We performed further subgroup analysis in patients receiving adequate BCG usage. It remained significant differences in terms of CCI (*p* < 0.01), the proportion of patients with hypertension (*p* < 0.01), and hyperlipidemia (*p* < 0.01) between the four subgroups. Gender also remained similar between groups (Supplementary Table 3).

**Fig. 1:**
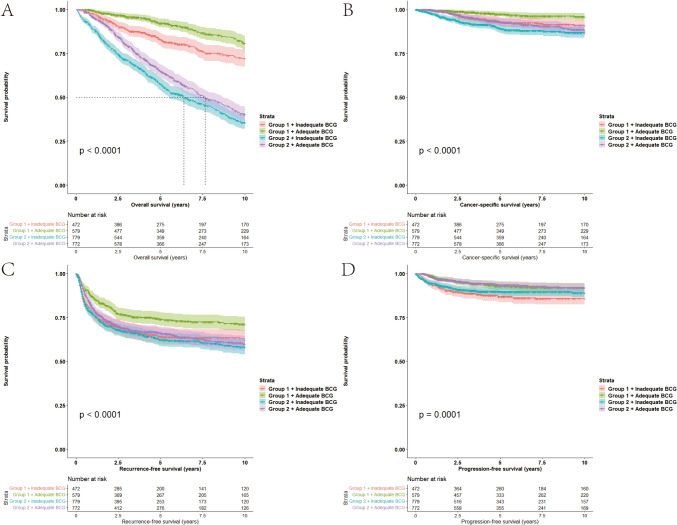
Kaplan Meier curves for Survival outcomes upon subgroup analysis. **A**) OS, **B** CSS, **C** RFS, **D** PFS.

On Kaplan–Meier analysis, there were significant differences between groups in OS (*p* < 0.0001), CSS (*p* < 0.0001), RFS (*p* < 0.0001), and PFS (*p* = 0.0001) (Fig. 1). The pairwise comparison (Benjamini–Hochberg method) of the survival outcomes of subgroups were performed (Supplementary Table 4). Multivariable Cox regression showed that younger patients who had adequate BCG treatment was superior to older with inadequate BCG in OS (HR: 0.20, 95% CI 0.16–0.26, *p* < 0.001), CSS (HR: 0.27, 95% CI 0.16–0.46, *p* < 0.001), RFS (HR: 0.64, 95% CI 0.52–0.78, *p* < 0.001), and PFS (HR: 0.58, 95% CI 0.39–0.87, *p* < 0.01). When compared with those older patients who received inadequate BCG treatment, younger patients who received inadequate BCG treatment had better OS (HR: 0.35, 95% CI 0.28–0.43, *p* < 0.001), and CSS (HR: 0.63, 95% CI 0.41–0.96, *p* = 0.03), while older patients who received adequate BCG treatment was associate with better OS (HR: 0.83, 95% CI 0.72–0.95, *p* < 0.01), and PFS (HR: 0.59, 95% CI 0.41–0.86, *p* < 0.01) (Table 2).

**Table 2:** Multivariable Cox regression analysis of subgroups

Variables	Overall survival		Cancer-specific survival		Recurrence-free survival		Progression-free survival	
	HR (95%CI)	*p* value	HR (95%CI)	*p* value	HR (95%CI)	*p* value	HR (95%CI)	*p* value
*Grouping*								
Group 2 + Inadequate BCG	Ref		Ref		Ref		Ref	
Group 2 + Adequate BCG	**0.83 (0.72–0.95)**	< 0.01	0.72 (0.51–1.03)	0.07	0.90 (0.75–1.06)	0.20	**0.59 (0.41–0.86)**	< 0.01
Group 1 + Inadequate BCG	**0.35 (0.28–0.43)**	< 0.001	**0.63 (0.41–0.96)**	0.03	0.94 (0.77–1.15)	0.55	1.17 (0.82–1.66)	0.40
Group 1 + Adequate BCG	**0.20 (0.16–0.26)**	< 0.001	**0.27 (0.16–0.46)**	< 0.001	**0.64 (0.52–0.78)**	< 0.001	**0.58 (0.39–0.87)**	< 0.01
*Gender*								
Female	Ref		Ref		Ref		Ref	
Male	**1.35 (1.15–1.59)**	< 0.001	1.04 (0.73–1.49)	0.83	**1.24 (1.04–1.47)**	0.02	**1.91 (1.29–2.85)**	< 0.01
*Charlson Comorbidity Index*								
2	Ref		Ref		Ref		Ref	
3	**1.46 (1.25–1.71)**	< 0.001	0.98 (0.68–1.43)	0.93	1.02 (0.86–1.21)	0.79	0.81 (0.57–1.15)	0.24
4	**2.08 (1.69–2.57)**	< 0.001	1.17 (0.69–1.99)	0.55	0.95 (0.74–1.21)	0.65	0.91 (0.55–1.51)	0.71
5–10	**2.71 (2.16–3.39)**	< 0.001	1.21 (0.64–2.28)	0.55	1.03 (0.77–1.38)	0.82	0.83 (0.44–1.58)	0.57
*Hypertension*								
No	Ref		Ref		Ref		Ref	
Yes	1.02 (0.88–1.18)	0.79	1.16 (0.83–1.63)	0.39	**1.18 (1.01–1.38)**	0.04	0.99 (0.73–1.35)	0.96
*Hyperlipidemia*								
No	Ref		Ref		Ref		Ref	
Yes	**0.70 (0.60–0.83)**	< 0.001	0.69 (0.46–1.04)	0.08	0.88 (0.74–1.05)	0.17	0.79 (0.54–1.16)	0.23

## Discussion

Using one of the largest cohorts of NMIBC patients treated with BCG, our study demonstrates that age influences most of the survival outcomes (OS, CSS, RFS) of NMIBC patients treated with adjuvant BCG following TURBT. When further investigating the impact of adequate BCG treatment, younger patients treated with adequate BCG had the best survival results (OS, CSS, RFS, and PFS), while the older ones treated with inadequate BCG had the worst OS, and CSS. Similar PFS was observed regardless of age group, as long as adequate BCG was given. All these phenomena led to the strong recommendation of adequate BCG treatment for eligible patients of all ages and further support the underlying hypothesis that immunosenescence can play a pivotal role in the response of elderly to BCG treatment.

Age is an important factor which influences treatment outcomes and prognosis of cancer, chronic disease, and mortality [14]. Within the uro-oncology field, it appears to be a prediction risk factor for kidney cancer [15], UTUC[16], prostate cancer[17], and bladder cancer[11, 18, 19].

Contieri et al. reported that older age (> 70 years old) did not affect progression rate or high-grade intravesical recurrence in a 632 patients’ cohort who had adequate BCG treatment [11]. Similar results were observed in our study, with adequate BCG treatment reducing 41% and 42% the risk of progression for both age groups. Another study conducted by Herr investigated the association between age groups and initial response to BCG, in terms of cancer-free survival (CFS)[18]. Controversially, patients aged 60 to 69 years had better CFS as compared to their younger counter part (50 to 59 years old); however, when using 70 years old as cut-off, Herr found older patients had worsen CFS, which was in line with our RFS results.

Although not fully comparable as it also includes low-risk patients, Oddens et al. compared the impact of age on prognosis in stage Ta or T1 bladder cancer patients treated with intravesical maintenance BCG immunotherapy or maintenance chemotherapy with epirubicin. The results demonstrated that epirubicin was inferior to BCG irrespective of patient age. However, a shorter time to progression was observed in older BCG patients (> 70 years old). These results differ from our observations, potentially due to bias in the analytical samples included in the study. Since different study population had been reported [19–21], the randomization process of EORTC Genito-Urinary Group Study 30,911 itself introduced bias when stratified age group were analyzed.

The mechanism of action of intravesical BCG immunotherapy is thought to be the result of the combined action of urothelial cells (including bladder cancer cells themselves) and cells of the immune system [6]. Therefore, immunosenescence may play a crucial role in the lower therapeutic antitumour effect of BCG. Although it is a logical hypothesis, this has not yet found scientific demonstration. Ershler et al. suggested that senescent host factors hindering the growth and spread of bronchial tumours in elderly patients [22]. In breast cancer, Fisher et al. found less aggressive features of breast tumour in elderly patients from a retrospective study of 1,869 women [23]. Recently, Gödde et al. investigate age-related degenerative changes in the pelvic lymph nodes from 390 prostate cancer patients [24]. The size of lymph nodes, capsular fibrosis, lipomatous atrophy, framework fibrosis, and calcifications were measured semi-quantitatively and compared between ≤ 60, 61 to 70, and ≥ 71 years patients. With increasing age, size of lymph nodes decreased, but capsular fibrosis, lipomatous atrophy, framework fibrosis, and calcifications increased. These might explain why younger patients treated with inadequate BCG in our cohort suffer from worse PFS. Despite the immunosenescence, adequate BCG may still activate the immune system strong enough to prevent progression as we observed patients with different age had almost the same PFS benefits.

There are several limitations in our study. First, our BCG patients and their medical conditions were retrieved primarily by diagnostic codes: although the same electronic system was used in all public hospitals in Hong Kong, there could be missing data and the data accuracy can be affected. Second, some data such as the histological reports and imaging results could not be retrieved through the electronic database. Recurrence was defined by the need of TURBT, and progression event was defined by the need of cystectomy, radiotherapy, or initiation of systemic treatment. However, these assumptions are prone to error, and the data accuracy might be affected. Third, despite the heterogeneity within the inadequate BCG subgroup, we are unable to make a more detailed comparison. Despite these limitations, this is by far the largest BCG cohort with long-term survival data, and we do believe our study provide important information regarding the efficacy of BCG in the long-run.

## Conclusion

Younger NMIBC patients (< = 70 Years old) showed better OS, CSS, RFS, but not PFS. Older Patients (> 70 years old) treated with adequate BCG treatment had similar PFS as their younger counterpart. Thus, in terms of PFS benefit, patients of all ages are strongly recommended to have adequate BCG treatment.

## Supplementary Information

Below is the link to the electronic supplementary material.

Supplementary file1 (DOCX 264 KB)

## Data Availability

Individual, de-identified participant data used in these analyses will be shared on request from any qualified investigator after the approval of a protocol and signed data access agreement via The Chinese University of Hong Kong (Hong Kong, China).
