# Supplementary material for: Young age and adequate BCG are key factors for optimal BCG treatment efficacy in non-muscle-invasive bladder cancer
Source: World J Urol. 2024 Sep 27;42(1):547. doi: 10.1007/s00345-024-05218-4 (PMC11436433; doi:10.1007/s00345-024-05218-4)
Supplement: Supplementary file 1 — Supplementary file1 (DOCX 264 KB) [file 345_2024_5218_MOESM1_ESM.docx]

Supplementary Figure 1. Inclusion and exclusion criteria, Cohort definition.


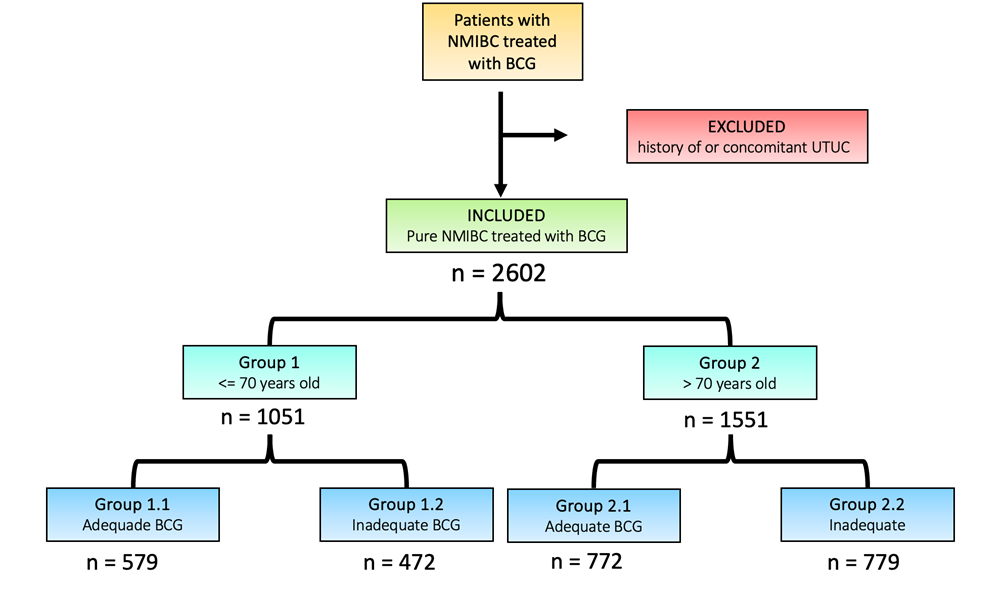


Supplementary Figure 2. Kaplan Meier curves for Survival outcomes. A) OS, B) CSS, C) RFS, D) PFS.


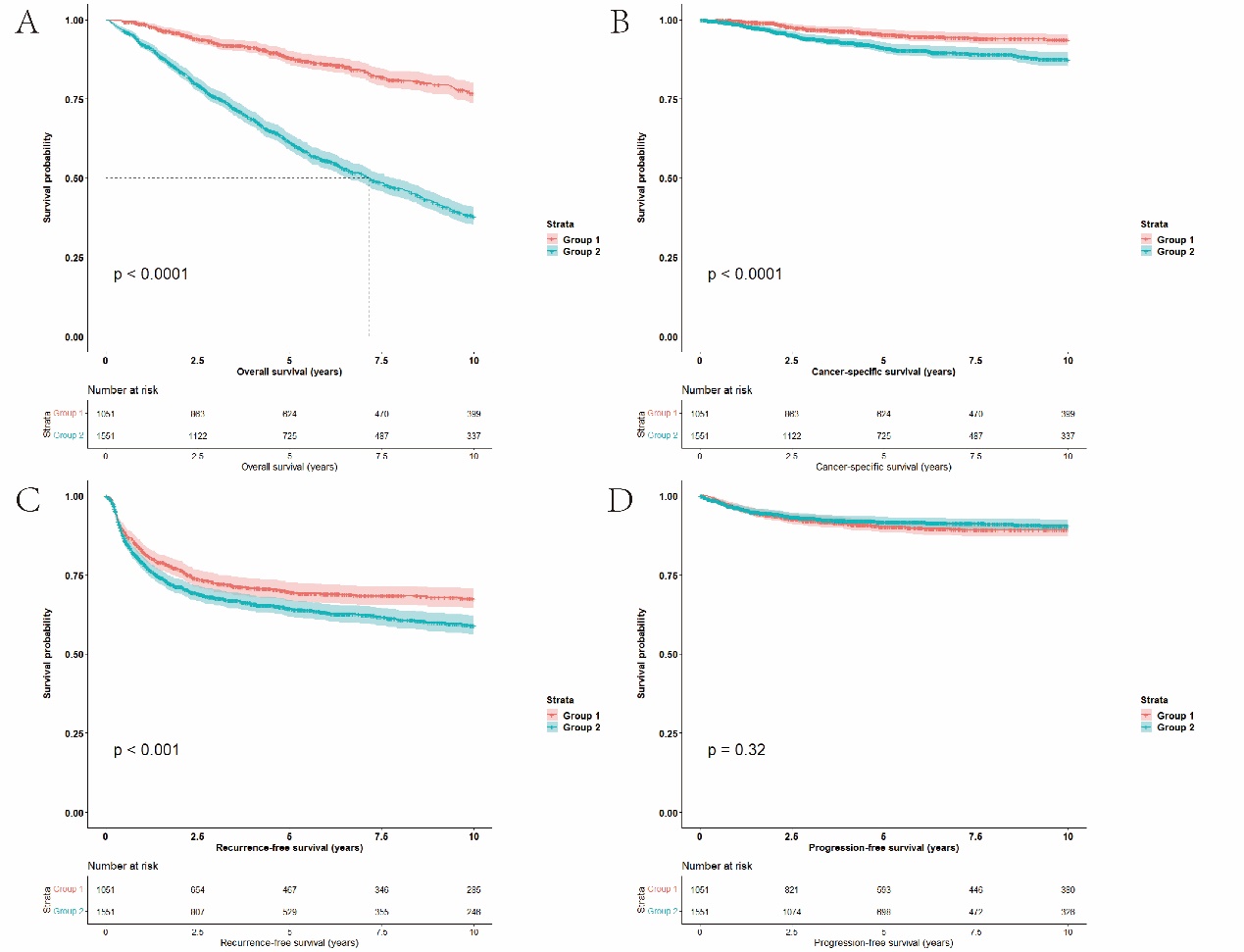


Supplementary Table 1. ICD-9-CM diagnosis codes of comorbidities.

Supplementary Table 2. Baseline characteristics between group 1(<=70y) and group 2 (>70y).

| Characteristics | Group 1 (<=70y) n = 1,051 | Group 2 (>70y) n = 1,551 | *p value* |
| --- | --- | --- | --- |
| Adequate BCG, n (%) |  |  |  |
| No | 472 (44.9) | 779 (50.2) | 0.01 |
| Yes | 579 (55.1) | 772 (49.8) |  |
| Gender, n (%) |  |  |  |
| Female | 212 (20.2) | 342 (22.1) | 0.25 |
| Male | 839 (79.8) | 1209 (77.9) |  |
| Charlson Comorbidity Index, n (%) | |  |  |
| 2 | 723 (68.8) | 733 (47.3) | <0.01 |
| 3 | 209 (19.9) | 462 (29.8) |  |
| 4 | 74 (7.0) | 210 (13.5) |  |
| 5-10 | 45 (4.3) | 146 (9.4) |  |
| Hypertension |  |  |  |
| No | 668 (63.6) | 573 (36.9) | <0.01 |
| Yes | 383 (36.4) | 978 (63.1) |  |
| Hyperlipidemia |  |  |  |
| No | 826 (78.6) | 1058 (68.2) | <0.01 |
| Yes | 225 (21.4) | 493 (31.8) |  |

Supplementary Table 3. Baseline characteristics between subgroups.

| Characteristics | Group 1 +  Inadequate BCG n = 472 | Group 1 +  Adequate BCG n = 579 | Group 2 +  Inadequate BCG n = 779 | Group 2 +  Adequate BCG n = 772 | *p value* |
| --- | --- | --- | --- | --- | --- |
| Gender, n (%) |  |  |  |  |  |
| Female | 99 (21.0) | 113 (19.5) | 171 (22.0) | 171 (22.2) | 0.65 |
| Male | 373 (79.0) | 466 (80.5) | 608 (78.0) | 601 (77.8) |  |
| Charlson Comorbidity Index, n (%) | |  |  |  |  |
| 2 | 318 (67.4) | 405 (69.9) | 354 (45.4) | 379 (49.1) | <0.01 |
| 3 | 97 (20.6) | 112 (19.3) | 237 (30.4) | 225 (29.1) |  |
| 4 | 35 (7.4) | 39 (6.7) | 109 (14.1) | 101 (13.1) |  |
| 5-10 | 22 (4.6) | 23 (4.1) | 79 (10.1) | 67 (8.7) |  |
| Hypertension |  |  |  |  |  |
| No | 384 (81.4) | 442 (76.3) | 552 (70.9) | 506 (65.5) | <0.01 |
| Yes | 88 (18.6) | 137 (23.7) | 227 (29.1) | 266 (34.5) |  |
| Hyperlipidemia |  |  |  |  |  |
| No | 307 (65.0) | 361 (62.3) | 295 (37.9) | 278 (36.0) | <0.01 |
| Yes | 165 (35.0) | 218 (37.7) | 484 (62.1) | 494 (64.0) |  |

Supplementary Table 4. The p value of pairwise comparison analysis of the survival, Benjamini-Hochberg method.

|  | OS | CSS | RFS | PFS |
| --- | --- | --- | --- | --- |
| >70 yrs + Adequate BCG vs. >70 yrs + Inadequate BCG | <0.01 | 0.06 | 0.28 | 0.02 |
| ≤70 yrs + Inadequate BCG vs. >70 yrs + Inadequate BCG | <0.0001 | 0.04 | 0.41 | 0.17 |
| ≤70 yrs + Adequate BCG vs. >70 yrs + Inadequate BCG | <0.0001 | <0.0001 | <0.0001 | 0.03 |
